# Timing of therapy for latent tuberculosis infection among immigrants presenting to a U.S. public health clinic: a retrospective study

**DOI:** 10.1186/1471-2458-8-158

**Published:** 2008-05-12

**Authors:** Kathleen R Page, Yukari C Manabe, Akintoye Adelakun, Lynn Federline, Wendy Cronin, James D Campbell, Susan E Dorman

**Affiliations:** 1 Department of Medicine, Johns Hopkins University School of Medicine, Baltimore, MD, USA; 2 Prince Georges County Tuberculosis Control Program, Cheverly, MD, USA; 3 Maryland State Department of Health and Mental Hygiene, Baltimore, MD, USA; 4 Department of Pediatrics, University of Maryland School of Medicine, Baltimore, MD, USA

## Abstract

**Background:**

In the U.S. more than half of incident tuberculosis (TB) cases occur in immigrants. Current guidelines recommend screening and treatment for latent TB infection (LTBI) within 5 years of arrival to the U.S. This study evaluates the timing of LTBI therapy among immigrants presenting for care to a public health TB clinic.

**Methods:**

Retrospective chart review of patients prescribed LTBI treatment based on medical records from Prince Georges County Health Department.

**Results:**

1882 immigrants received LTBI therapy at Prince Georges County Health Department between 1999 and 2004. 417 of these patients were diagnosed with LTBI through contact investigations and were excluded from the analysis. Among the remaining 1465 individuals, median time from arrival to the U.S. until initiation of LTBI therapy was 5 months (range 0–42.4 years). 16% of all immigrants initiated therapy more than 5 years after arrival to the U.S. A logistic regression model using risks identified on univariate analysis revealed that referral for therapy by non-immigration proceedings was the strongest predictor of initiation of therapy more than 5 years after arrival to the U.S. Other factors associated with > 5 year U.S. residence prior to initiation of LTBI therapy included female gender (adjusted odds ratio (AOR) 1.8, 95% CI 1.2–2.6), age ≥ 35 (AOR = 4.1, 95% 2.5–6.6), and originating from Latin American and the Caribbean (AOR = 1.9, 95% CI 1.3–3.0).

**Conclusion:**

Foreign-born individuals who are not referred for LTBI therapy through immigration proceedings are less likely to receive LTBI therapy within 5 years of arrival to the U.S. These data highlight the need to explore other mechanisms for timely LTBI screening beyond services provided by immigration.

## Background

Tuberculosis (TB) case rates in the United States have declined since 1993, but the decline among foreign-born persons has been less substantial than that among individuals born in the U.S.[1] Each year since 2002, more than half of incident TB cases in the U.S. occurred among foreign-born individuals[1]. In order to accelerate the decline of TB in the U.S., the Institute of Medicine has emphasized the need to identify and treat individuals with latent TB infection (LTBI), including the need for screening of immigrants from countries with high TB rates[2]. A key component of the U.S. TB elimination strategy is the identification and treatment of foreign-born individuals at high risk for TB [3,4]. Current U.S. immigration laws require screening persons older than 14 years old for active TB with a chest radiograph during the application for immigrant visas prior to entry to the U.S. Individuals with abnormal radiographs suggestive of TB have three sputum smears evaluated for the presence of acid fast bacilli. Immigrants with abnormal radiographs but no evidence of active TB are able to travel to the U.S. but are required to report to a local health department for further evaluation within 30 days of arrival to the U.S. Many of these patients are found to be candidates for LTBI therapy[5]. Screening for LTBI with the tuberculin skin test is also required for immigrants already residing in the U.S. who apply for adjustment of immigrant status to permanent residents (Code of Federal Regulations, Title 42) [6,7].

Therefore, LTBI screening among foreign-born populations targets individuals at high risk of TB applying for entry visas to the U.S. or immigrants already residing in the U.S. applying for permanent residency. This strategy facilitates prompt recognition of TB disease in some classes of documented immigrants at risk for TB [5,8-10], but does not address the needs of people from TB endemic areas who enter the U.S. without documentation or who overstay tourist visas and do not apply for status change [3,10]. Screening and treatment for latent infection are recommended for all foreign-born persons who have resided in the U.S. for up to 5 years [3,11], since the highest rates of active disease occur in this group. Although the risk of active TB among foreign-born individuals who have lived in the U.S. for more than 5 years is lower than in recent arrivals, rates in this population are significantly higher than in U.S. born individuals[12].

Because LTBI is asymptomatic and screening programs are uncommon outside the U.S., individuals at high risk for TB who are not screened through immigration proceedings may not seek LTBI testing on their own. A key question for TB control is how to expand timely LTBI screening programs for this population. In this study, we evaluate the characteristics of foreign-born patients presenting to a public health clinic for LTBI therapy and the timing of presentation relative to the time of arrival to the U.S. Characterization of patients that initiate therapy can identify successful interventions and provide an insight into strategies that require further attention.

## Methods

We performed a retrospective study of all foreign-born individuals treated for LTBI during the period of January 1, 1999 to January 31, 2004 at the Prince Georges County Health Department in Maryland using an existing database[13]. In brief, a list of all patients who were treated for LTBI during the study period was obtained from a computerized registration database maintained by the TB control program. Information was obtained by medical record review, abstracted onto a data form, and entered into a study database. We also obtained non-identified information from the registration database regarding the country of origin of patients receiving LTBI therapy from 1990–2004. Institutional review boards of Johns Hopkins University School of Medicine and Maryland Department of Health and Mental Hygiene approved this study.

Data obtained included: gender, age, self-identified race/ethnicity, country of origin, date of U.S. arrival, date of first clinic visit, reason for LTBI therapy, source of referral, prescribed LTBI treatment regimen, tuberculin skin test size, prescribing clinician, adverse drug reactions, completion of therapy, and HIV serostatus. For analytical purposes, two age categories (≤ 35, > 35 years), and four race/ethnicity categories (White non-Hispanic, Black non-Hispanic, Hispanic & Asia/Other) were used. Native Hawaiians and Pacific Islanders were included in the Asia/Other category due to low numbers (n = 5). The four categories for region of origin (Europe & Canada, Latin America & the Caribbean, Africa, and Asia) were derived from country of origin data and were chosen to reflect regional similarities in the risk of tuberculosis and immigration patterns. Source of referral was categorized into Immigration, Primary Medical Doctor, and Other (self-referrals and walk-ins).

All persons with LTBI referred for treatment and not known to have recent contact with an infectious TB case were assumed to have been infected in their countries of origin. The primary outcome was duration of time between arrival to the U.S. and initiation of LTBI therapy. This outcome was calculated by subtracting the date of first visit to the clinic from self-reported date of arrival to the U.S. and categorized into 3 groups (< 1 year, 1–5 years and > 5 years) for analysis. "LTBI therapy delay" was defined as LTBI initiated > 5 years after arrival since current LTBI screening and treatment guidelines do not target immigrants who have lived in the U.S. more than 5 years [11].

Univariate associations between predictors and time to referral were examined using Chi square for categorical variables (gender, age category, race/ethnicity, region of origin, reason for LTBI therapy, HIV serostatus, source of referral), and Wilcoxon rank-sum test for non-normally distributed continuous variables (age, TST size). Univariate predictors showing a statistical association with delay (p < 0.05) were entered into the multivariate model. The final model was derived by removing insignificant (p > 0.05) variables as determined by the likelihood ratio test. Race/ethnicity was removed from the final model due to collinearity with region of origin, as measured by the variance inflation factor. Adjusted odds ratios (AOR) and corresponding 95% confidence intervals (95% CI) were calculated for the variables that remained in the final model. Analyses were performed using STATA Statistical Software (Stata Corp: Stata Statistical Software: Release 8.0, Stata Corporation, College Station, TX, 2002).

## Results

### Baseline characteristics of the study population

A total of 1882 foreign-born patients were offered LTBI therapy at Prince George's County TB clinic from January 1, 1999 to January 21, 2004. In order to minimize the possibility of including individuals infected with TB after arrival to the US, we excluded 417 individuals who were diagnosed with LTBI during a contact investigation because we could not rule out the possibility of recently acquired infection. In addition, 3 patients had incomplete data and were excluded from the final multivariate model which included 1463 patients. Baseline characteristics of the study population are shown in Table 1. The cohort was relatively young (median age 23 yrs, interquartile range or IQR = 15–32) with a slight female predominance (54%). The vast majority of patients (n = 1406, 96%) originated from one of 3 regions: Africa (n = 839, 57%), Latin America & the Caribbean (n = 414, 28%), and Asia (n = 153, 11%). Relatively few patients were born in Europe & Canada (n = 53, 4%). Racial and ethnic distribution mimicked the distributions of the region of origin. Among patients tested for HIV (n = 508), few were HIV seropositive (n = 10, 2%). Of the patients with unknown HIV status (n = 957), one fifth (n = 180, 19%) refused HIV testing, and the remainder (n = 777, 81%) had no documentation of HIV counseling or testing. A greater proportion of patients from Latin America & the Caribbean (92%) or Asia (66%) had an unknown HIV serostatus compared to patients from Africa (54%) or Europe & Canada (30%, p = 0.001). However, refusal of HIV testing was not associated with region of origin (Chi square, p = 0.96).

**Table 1:** Baseline characteristics according to length of U.S. residence at initial visit for LTBI therapy

***Length of U.S. Residence***						
	***Total (n, %)***	***Median Time in Months (IQR)***	***< 1 year (n, %)***	***1–5 years (n, %)***	***> 5 years (n, %)***	***p value***
**Total**	1465	5 (2–30)	899 (61.4)	336 (22.9)	230 (15.7)	
**Gender**						
Male	673 (46.0)	4 (2–24)	448 (66.6)	140 (20.8)	85 (12.6)	< 0.001
Female	789 (54.0)	7 (0.2–77)	449 (59.9)	195 (24.7)	145 (18.4)	
**Age**						
Median (IQR)	23 (15–32)		20 (14–30)	24 (17–32)	30 (24–36)	< 0.001
< 35 yrs	1180 (80.6)	5 (2–28)	741 (62.8)	277 (23.5)	162 (13.7)	
≥ 35 yrs	285 (19.5)	5 (2–54)	158 (55.4)	59 (20.7)	68 (23.9)	
**Region of Origin**						
Africa	839 (57.4)	4 (2–14)	604 (72.0)	170 (20.3)	65 (7.8)	< 0.001
Latin America & Caribbean	414 (28.3)	28 (6–79)	140 (33.8)	138 (33.3)	136 (32.9)	
Asia	153 (10.7)	4 (2–26)	102 (66.7)	24 (15.7)	27 (17.7)	
Canada & Europe	56 (3.8)	2 (1–4)	51 (91.1)	3 (5.4)	2 (3.6)	
**Race/Ethnicity**						
White	59 (4.0)	2 (1–4)	53 (89.8)	4 (6.8)	2 (3.4)	< 0.001
Hispanic	352 (24.1)	25 (6–72)	129 (36.7)	119 (33.8)	104 (29.6)	
Black	876 (59.9)	4 (2–18)	603 (68.8)	180 (20.6)	93 (10.6)	
Other	175 (12.0)	4 (2–170)	113 (64.6)	32 (18.2)	30 (17.1)	
**TST size (mm)**						
Size (IQR)	15 (12–20)		15 (12–19)	15 (13–20)	16 (14–20)	0.67
**HIV status**						
Negative	498 (34.0)	2 (1–4)	440 (88.3)	30 (6.0)	28 (5.6)	< 0.001
Positive	10 (0.7)	10 (4–16)	5 (50.0)	3 (30.0)	2 (20.0)	
Unknown	957 (65.3)	13 (4–49)	454 (47.4)	303 (31.7)	200 (20.9)	
**Referral**						
Immigration	666 (46)	2 (1–4)	638 (95.8)	24 (3.6)	4 (0.6)	< 0.001
Primary Medical Doctor	121 (8.4)	35 (12–90)	28 (23.1)	48 (39.7)	45 (37.2)	
Other*	662 (45.7)	24 (7–64)	226 (34.1)	259 (39.1)	177 (26.7)	

* Other = self-referrals, walk-in patients

### Time to initiation of LTBI therapy after arrival to the U.S

Table 1 shows the period of residence in the U.S. in years at initiation of LTBI therapy according to baseline patient characteristics. The median time from arrival to the U.S. until LTBI therapy was 5 months (IQR range 2–30 months). In accordance to guidelines targeting recent arrivals to the U.S., the majority of patients received LTBI therapy within 5 years of arrival to the U.S. (n = 1235, 84%); 16% of the patients had been in the U.S. for over 5 years prior to receiving LTBI therapy (n = 230).

Univariate analysis identified the following risk factors for initiation of LTBI therapy more than five years after arrival to the U.S.: age, gender, region of origin, race/ethnicity, HIV status, and source of referral (Table 1). In multivariate analysis adjusting for gender, age, region of origin, HIV serostatus, and source of referral we identified source of referral as the major predictor for delays in LTBI therapy (Table 2). Patients who were not referred for LTBI therapy through immigration proceedings had a very high risk of receiving LTBI therapy more than 5 years after arrival to the U.S. More than one third of patients referred for LTBI therapy by their primary medical doctor (PMD) received therapy 5 years after arrival to the U.S. (AOR = 251.7, 95% CI 78.4–808.2), and over one fourth of patients who self-referred or walked-in to the TB clinic received LTBI therapy 5 years after arrival (AOR = 113.7, 95% CI 38.5–335.6). Other risk factors for delayed LTBI therapy included female gender (AOR = 1.80, 95% C.I. 1.24–2.60), age > 35 years old (AOR = 4.11, 95% C.I. 2.54–6.64), and originating from Latin America and the Caribbean (AOR = 1.95, 95% C.I. 1.28–2.95)

**Table 2:** Factors associated with initiation of LTBI therapy more than 5 years after arrival to the U.S.

	**Length of U.S. Residence**	
	**1 – 5 years**	**> 5 years**

	AOR* (95% CI)	AOR* (95% CI)

**Gender**		
Male	Reference	
Female	1.3 (1.0–1.8)	1.8 (1.2–2.6)
		
**Age**		
< 35	Reference	
≥ 35	1.9 (1.2–3.0)	4.1 (2.5–6.6)
		
**Region of Origin**		
Africa	Reference	Reference
Latin America & Caribbean	0.7 (0.5–1.0)	1.9 (1.3–3.0)
Asia	0.6 (0.3–1.0)	1.7 (0.9–3.2)
Canada & Europe	0.6 (0.1–2.4)	1.4 (0.2–7.5)
		
**HIV Status**		
Negative	Reference	
Positive	1.2 (0.2–5.7)	0.6 (0.1–3.5)
Unknown	2.4 (1.5–4.0)	1.1 (0.6–1.9)
		
**Referral**		
Immigration	Reference	Reference
Primary Medical Doctor	38.3 (19.6–74.6)	251.7 (78.4–808.2)
Other**	24.8 (14.9–41.2)	113.7 (38.5–335.6)

Abbreviations: AOR adjusted odds ratio, CI = confidence interval AOR obtained after adjustment for gender, age, region of origin, and HIV status

* Reference category for outcome is < 1 year

** Other = self-referrals, walk-in patients

**Figure 1:**
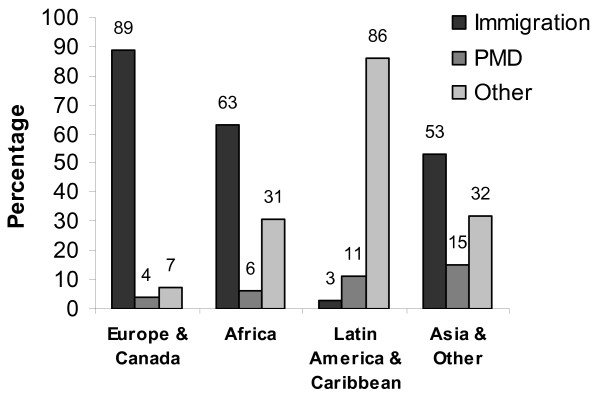
**Source of referral for LTBI therapy in our study cohort according to region of origin, Prince Georges County Health Department, Maryland, 1999–2004.** Abbreviation: PMD = Primary Medical Doctor. Immigration = Referral from the U.S. Citizenship and Immigration Services following medical evaluation for refugees, immigrants, and status adjustment.

Given the strong association between timing of LTBI therapy and source of referral, we stratified source of referral by region of origin, age, and gender. Figure 1 shows that more than half of the patients from Africa (63%), Asia (53%), and Europe and Canada (89%) were referred through immigration proceeding related to applications for refugees, immigrants, and status adjustment, compared to only 3% of patients from Latin America and the Caribbean. Eleven percent of patients from Latin America & the Caribbean were referred by their primary medical doctor (PMD), but over 80% of patients from this region self-referred or walked-in to the clinic. Univariate analysis showed that fewer women than men (43% vs. 50%, Chi square p = 0.016) were referred to LTBI therapy by immigration services, but there was no significant association between age and source of referral (Chi square p = 0.41). Multivariate analysis adjusting for age, gender, and region of origin showed that region of origin was the only factor independently associated with referral by immigration proceedings (Table 3).

**Table 3:** Factors associated with source of referral

	**Immigration **n, %	**PMD **n, %	**Other **n, %	AOR*
**Total**	666 (46.0)	121 (8.4)	662 (45.7)	
**Gender**				
Male	334 (50.0)	55 (8.2)	279 (41.8)	Reference
Female	332 (42.7)	65 (8.4)	381 (49.0)	0.9 (0.6–1.4)
**Age**				
< 35 yrs	527 (45.1)	97 (8.3)	543 (46.5)	Reference
≥ 35 yrs	139 (49.3)	24 (8.5)	119 (42.2)	1.4 (0.8–2.5)
**Region of Origin**				
Africa	524 (63.1)	50 (6.0)	256 (30.8)	Reference
Latin America & Caribbean	12 (2.9)	47 (11.5)	350 (85.6)	0.02 (0.01–0.05)
Asia	80 (53.0)	22 (14.6)	49 (32.4)	0.3 (0.2–0.6)
Canada & Europe	50 (89.3)	2 (3.6)	4 (7.1)	2.3 (0.6–9.9)

AOR = Adjusted odds ratio for referral through immigration proceedings

Complete data available for 1443 individuals

### Regional distribution and source of referral for patients treated for LTBI at Prince George's County Health Department

**Figure 2:**
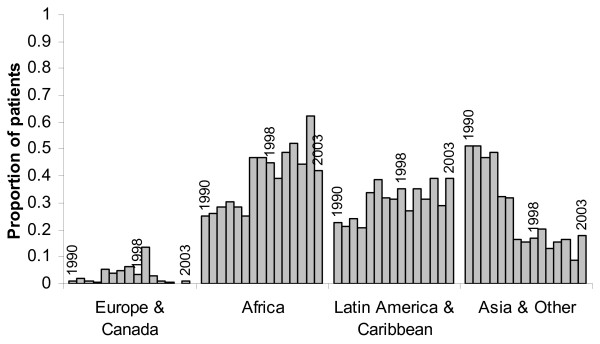
Regional Distribution of Foreign-born Individuals Receiving LTBI Therapy at Prince Georges County Health Department, Maryland, 1990–2004.

In order to determine whether the regional differences in time to initiation of LTBI therapy after arrival to the U.S. could be due to a cohort effect, we obtained non-identified information regarding region of origin from the computerized registration database for all foreign-born individuals treated for LTBI from 1990–2004. Figure 2 shows the proportion of immigrants from Asia, Africa, Latin America & the Caribbean, and Europe & Canada who received LTBI therapy at Prince Georges County since 1990. These data suggest that immigration to Prince Georges County from all regions began more than 5 years prior to our dates of inclusion in our database (1998–2004).

## Discussion

In this retrospective study of 1465 foreign-born individuals receiving LTBI therapy at a public TB clinic, we found that referral through immigration proceedings was the strongest predictor of timely initiation of LTBI therapy. In fact, 99.4% of patients referred by immigration proceedings received LTBI therapy within 5 years of arrival to the U.S. as recommended by current guidelines [11]. These findings are not surprising given the close link between TB control programs and immigration proceedings. However, a significant proportion of foreign-born individuals in the U.S. never seeks entry visas and may not have access to immigration-based LTBI screening programs. It is estimated that over 25% of immigrants currently living in the U.S. are undocumented and are not in a position to apply for permanent residency status [14].

In our study, over 50% of patients were not referred through immigration proceedings. These patients were at high risk for receiving LTBI therapy more than 5 years after arrival to the U.S. This finding is concerning because guidelines published in 2000 by the Centers of Disease Control and Prevention (CDC) and the American Thoracic society recommend testing and treating only foreign-born persons who have been in the U.S. for 5 years or less[11]. However, immigrants have a high risk of TB even after living in the U.S. for more than 5 years and would likely benefit from LTBI therapy. In 2004, almost one quarter (24%) of all cases of TB in the U.S. occurred among immigrants who had arrived to the U.S. more than 5 years previously[12]. Over 50% of TB cases in Mexican-born individuals occurs after prolonged (> 5 years) duration of residence in the U.S.[15] Furthermore, DNA fingerprinting analysis of *M. tuberculosis *isolated from TB cases in foreign-born patients shows nonclustering, suggesting that most cases are due to reactivation of infection acquired in their country of origin[16,17]. These data suggest that timely diagnosis of LTBI has the potential to prevent most cases of active TB disease among immigrants who comply with therapy[18]. Adherence to LTBI therapy is almost twice as high among individuals from Latin America, the Caribbean and Asia compared to US-born individuals[13].

Other factors associated with delays in LTBI therapy included older age, female gender, and originating from Latin America and the Caribbean. Delays in adults older than 35 years might reflect previous guidelines which did not recommend LTBI therapy for patients older than 35 years, discouraging LTBI testing in this age group[19]. However, this finding is subject to bias or cohort effect since younger immigrants are less likely to have been in the U.S. for > 5 years. Our dataset did not allow us to discern why females may be at higher risk for delays in LTBI therapy, though gender inequalities have been documented for other preventive services. Although fewer women were referred by immigration proceedings, gender was not independently associated with referral mode. Almost one third of patients from Latin America and the Caribbean received LTBI therapy more than 5 years after arrival to the U.S. These findings are concerning given that in 2005 69% of all TB cases among foreign-born people in the U.S. occurred among individuals from Latin America and the Caribbean [20]. As in other immigrant groups, timing of LTBI therapy in this population was mostly influenced by referral mode since only 3% of patients from Latin America and the Caribbean were referred through immigration services. However, our data did not allow us to explore other independent risk factors associated with delays in LTBI therapy in this group.

Because our data were limited to patients who presented to the clinic for LTBI therapy, we could not evaluate the characteristics of immigrants who never access LTBI screening or therapy. Nonetheless, our findings provide a basis for future studies exploring other strategies for timely LTBI screening and therapy in this crucial population for TB control. Over 50% of the patients in our cohort were self-referred or referred by their PMD, highlighting the importance of developing LTBI programs beyond immigration service proceedings. Self-referrals and walk-ins were an important method of entry to the clinic, suggesting the increased awareness of public TB services at the community level may facilitate access to LTBI screening and therapy. Research is needed to identify factors associated with self-referral, such as personal acquaintances (word-of-mouth), educational programs, work-related requirements, or informational brochures. Raising awareness of LTBI screening among the medical community may also enhance timely referral for therapy in high risk populations. However, delayed referrals by primary medical providers may reflect poor access to general medical care in immigrant populations. Our data suggests that expansion of LTBI screening and treatment to include foreign-born individuals who have lived in the U.S. for more than 5 years may be one way to enhance TB control programs.

There are several limitations to our study. In estimating delays in initiation of LTBI therapy, we assumed that most LTBI infection in immigrants was acquired in the country of origin. However, epidemiologic clustering of active cases among immigrants from the same region may lead to ongoing exposure to TB after arrival in the U.S. We removed individuals identified through contact investigations to minimize the chance of including patients infected with TB after arrival to the U.S. Furthermore, despite the relatively large size of our patient population, our study was limited to one county TB control program in Maryland. The generalizability of our results to the total U.S. immigrant population depends on local immigration patterns and health care delivery strategies for foreign-born individuals.

## Conclusion

The CDC is considering several strategies to address the high rate of TB among foreign born individuals in the U.S., such as revising medical screening for applicants for U.S. immigration, and working with international organizations to reduce TB in the countries of origin[1]. These strategies should decrease the importation of new TB cases from other countries, but do not address the risk of TB in foreign-born individuals who already reside in the U.S., especially among undocumented immigrants who are not routinely screened at entry to the country. Our study shows that immigrants who are not referred by immigration proceedings have a high risk of presenting for LTBI therapy more than 5 years after arrival to the U.S. These data highlight the need for strategies beyond immigration-linked programs to identify and overcome missed opportunities for LTBI therapy among high risk foreign-born individuals, including those who have lived the U.S. for > 5 years.

## Competing interests

The authors declare that they have no competing interests.

## Authors' contributions

KRP participated in the design of the study, data acquisition, statistical analysis and drafting of the manuscript. YCM participated in the design of the study and drafting of the manuscript, AA participated in the design of the study and data acquisition, LF participated in the data acquisition, WC participated in the design of the study and statistical analysis, JDC participated in the statistical analysis and drafting of the manuscript, SED participated in the design of the study and drafting of the manuscript.

## Pre-publication history

The pre-publication history for this paper can be accessed here:


